# Antihypertensive Peptide ENWAAL Derived from *Coix* Glutelin and Its Effect on the Expression of SHR Renin–Angiotensin System

**DOI:** 10.3390/biom16060888

**Published:** 2026-06-16

**Authors:** Wenjing Zhang, Jinjie Liang, Yiping Li, Yong Yang, Haiying Chen, Liansheng Qiao, Lingzhi Wang

**Affiliations:** 1 School of Life Sciences, Beijing University of Chinese Medicine, Beijing 102488, China; 20230931205@bucm.edu.cn (W.Z.); 20210931202@bucm.edu.cn (J.L.); 20240931209@bucm.edu.cn (Y.L.); 20240931210@bucm.edu.cn (Y.Y.); 20230931206@bucm.edu.cn (H.C.); 2 School of Chinese Materia Medica, Beijing University of Chinese Medicine, Beijing 102488, China

**Keywords:** *Coix lacryma-joib* L., ACE, peptide, RAS, hypertension

## Abstract

Hypertension is one major risk factor of cardiovascular diseases, and RAS plays vital role during the development of hypertension. To obtain a novel antihypertensive peptide, *Coix* glutelin was hydrolyzed by trypsin and further separated by Sephadex G10. Based on 751 identified sequences, pharmacophore mapping, molecular docking, and in silico proteolysis were applied to screen and optimize the candidate sequence. Finally, a novel peptide, ENWAAL, was generated with IC_50_ of 210.57 μM, which acted with ACE in a competitively inhibitory pattern. The in vivo antihypertensive effect was evaluated in SHRs. Significant improvements were observed in hypertension-related characteristics, including blood pressure, cardiac structure and function, and serum angiotensin II (Ang II) level. In the brain, quantitative real-time PCR analysis revealed significant downregulation of angiotensin II type 1 receptor (*AT1R*) mRNA expression, concomitant with upregulation of angiotensin-converting enzyme 2 (*ACE2*) and MAS receptor. The protein expression of ACE and AT1R in the ENWAAL group also significantly decreased. This study can provide a candidate antihypertensive drug targeting RAS.

## 1. Introduction

Hypertension is one of the most common cardiovascular diseases in clinics that may cause adverse health consequences such as stroke, heart attack, heart failure, kidney damage, and numerous other complications [1]. According to the World Health Organization’s statistics, there are approximately 1.3 billion people with hypertension worldwide, and it causes up to 10 million deaths per year [2]. Therefore, this disease poses a significant public health concern.

The renin–angiotensin system (RAS) is a key regulator of blood pressure and fluid homeostasis [3]. In this system, the inactive angiotensin (Ang) I is catalyzed by angiotensin-converting enzyme (ACE) to yield the biologically active Ang II, which acts on Ang II type I receptor (AT1R) to cause diverse physiological effects, including vasoconstriction, cardiovascular hypertrophy, fibrosis, cellular oxidative stress damage [4], and disfunction of the vascular endothelial structure [5]. In contrast, the counterregulatory axis [ACE2/Ang-(1–7)/Mas receptor] mediates the opposite effects to those of Ang II, such as vasodilator, anti-hypertrophic, anti-fibrotic, and anti-inflammatory effects [6]. As a key enzyme in the synthesis of Ang II, ACE has become an important target of antihypertensive therapeutic, and ACE inhibitors (ACEIs) have been extensively utilized in clinical practice, such as captopril, lisinopril, etc. [7].

In addition to the “classical” circulating RAS, local or tissue RAS has been observed in diverse tissues. Among them, central RAS has attracted widespread attention, which was first proposed in 1961 [4]. Similar to its peripheral counterpart, brain RAS is important for controlling sympathetic tone and the release of endocrine factors that collectively determine blood pressure [8]. The central nervous system (CNS) network that regulates the sympathetic tone is mainly located in the brainstem and hypothalamus, in areas such as the rostral ventrolateral medulla (RVLM), hypothalamic paraventricular nucleus (PVN), subfornical organ (SFO), and nucleus tractus solitarius (NTS) [9]. Ang II can impair the cardiac baroreflex in the NTS via endothelia nitric oxide synthase (eNOS) [10] and activate the SFO-PVN-RVLM pathway to generate ROS, which are implicated in the pathogenesis of neurogenic hypertension [11]. In the Ang II-induced maternal hypertension model, adult male offspring exhibited upregulated RAS component expression in the lamina terminalis (LT) and PVN, key forebrain regions regulating cardiovascular and fluid homeostasis [12].

The commonly used antihypertensive drugs in clinical practice include ACEIs, angiotensin receptor blockers, calcium channel blockers, β-blockers, and thiazide and thiazide-like diuretics. These drugs confronted certain side effects, such as dry cough and angioedema [13]. Therefore, it is extremely urgent to develop safe and effective antihypertensive drugs that target both systemic RAS and central RAS.

*Coix lachryma-jobi* L., a useful herb in traditional Chinese medicine, has been historically employed for removing dampness and promoting diuresis, as documented in Shennong’s *Classic of Materia Medica* [14]. This herb contains diverse bioactive components, such as polysaccharides, proteins, esters, triterpenoids, and so on [15]. For instance, *Coix* seed oil (CSO) has gained significant attention for its great anticancer potential [16]. CSO also has emerged as a promising intervention for hyperuricemia due to its potential to alleviate oxidative damage and support organ health [17]. Polysaccharide of *Coix* seed has anti-inflammatory, antioxidation, hypoglycemic, immune regulation, anti-tumor, and other pharmacological effects [18]. *Coix* seed is rich in protein; therefore, the generation of bioactive peptides from *Coix* seeds has significant value in both food and medicine fields.

Previous studies have indicated that the hydrolysate of *Coix* glutelin possessed significant ACE inhibitory activity [19]. However, the peptide composition, in vivo antihypertensive efficacy, and effects on the central RAS remains elusive. In this study, chromatographic methods were applied to separate and purify the *Coix* seed glutelin hydrolysate, and the peptide sequences were subsequently identified. Furthermore, candidate peptides were structurally optimized after virtual screening. Finally, the in vivo antihypertensive activity and its effect on the key members’ expression in brainstem were investigated, including ACE, AT1R, ACE2, and Mas.

## 2. Materials and Methods

### 2.1. Materials

Seeds of *Coix lacryma-jobi* were purchased from Beijing Tong Ren Tang Pharmacy (Beijing, China). ACE was purchased from Sigma-Aldrich (St. Louis, MO, USA). The rat Ang II ELISA kit and bicinchoninic acid (BCA) assay kits were sourced from Beijing LABLEAD Trading Co., Ltd. The antibodies we used were as follows: anti-tubulin (F1566, Selleck, https://www.selleckchem.com/), anti-AT1R (bs-2132R, Beijing Bioss, Beijing, China), anti-ACE (sc-23908, Santa Cruz, https://www.scbt.com/zh/home), HRP-conjugated anti-mouse secondary antibody (RGAM001, Proteintech, Wuhan, China), and anti-rabbit secondary antibody (RGAR001, Proteintech, Wuhan, China).

### 2.2. Preparation of Enzymatic Hydrolysate of Coix Glutelin

The preparation of *Coix* glutelin was conducted according to the method of Li [20]. Briefly, storage protein fractions (albumin, globulin, prolamin, and glutelin) were sequentially extracted from the dried *Coix* powder. After the glutelin sample was freeze-dried, the samples were dissolved with deionized water at the material–liquid ratio of 1:100 (*w*/*v*), and trypsin was added to the reaction system with the enzyme–substrate ratio of 1:10 (*w*/*w*) subsequently. After 6 h of hydrolysis at 37 °C, the components ≤ 3 kDa were collected by ultrafiltration centrifugation, freeze-dried, and stored at −80 °C.

### 2.3. Separation of Peptides from Coix Hydrolysate

The hydrolysate with molecular ≤ 3 kDa was dissolved in deionized water and loaded onto a Sephadex G-10 gel filtration column (1.6 × 60 cm). The sample was eluted with deionized water at a flow rate of 1 mL/min and monitored at 220 nm. Fractions were collected at 10 min intervals.

### 2.4. Peptides Sequence Identification

The samples were analyzed using a nano-HPLC-MS/MS on an Easy-nLC 1000 system (Thermo fisher, USA), connected to an Orbitrap Fusion Lumos Tribrid-MS (Thermo fisher Scientific, Waltham, MA, USA), which was equipped with a Repro Sil-Pur C18-AQ column (15 cm × 0.15 mm, 1.9 μm, Dr Maisch, Germany). Mobile phase A was 0.1% formic acid solution, and mobile phase B was 80% acetonitrile in 0.1% formic acid solution. The sample was separated using a linear gradient eluted from 6% B to 95% B within 78 min, with a flow rate of 0.6 mL/min. The MS/MS parameters were set according to our previous study, with a charge-state of 2^+^~6^+^ and dynamic exclusion time of 20 s [19]. The data were analyzed using Proteome Discoverer 2.2 (Thermo Scientific, Waltham, MA, USA).

### 2.5. Pharmacophore Mapping of ACE Inhibitory Peptides

ACE inhibitory peptides are generally short-chained peptides [20]. Therefore, in this study, peptides containing 2–10 amino acids were selected as potent sequences for virtual screening. ACE pharmacophore was applied to predict the activity of the peptides. The 3D pharmacophore was constructed in our previous work [21], which contained four features, including a hydrogen bond donor, a hydrogen bond acceptor, a hydrophobic group, and a negatively charged group.

The 3D conformations of the peptides were constructed and energy-minimized using the CHARMm force field in Discovery Studio 4.0 (DS, Accelrys Inc., San Diego, CA, USA). In total, 255 conformations of peptides were generated for pharmacophore mapping, with the BEST mode and a relative energy threshold of 20.0 kcal mol^−1^. Fit value was the key index to evaluate the overlap between the candidate peptides and pharmacophore model. Peptides with fit value over 0.9 were selected for further analysis.

### 2.6. Molecular Docking and In Silico Proteolysis

The molecular docking was conducted to analyze the affinity and binding mode of ACE inhibitory peptides. The 3D structure of human ACE was obtained from PDB with ID numbers of 1O86, 4CA5, and 4BZR. Docking simulations were performed using CDOCKER (Discovery Studio 4.0).

To obtain peptides with higher ACE inhibitory activity, Expasy Peptide Cutter (Expasy—PeptideCutter) was used to conduct in silico proteolysis for selected peptides using pepsin (pH = 1.3), trypsin, and chymotrypsin high specificity.

### 2.7. Candidate Peptides Synthesis

Peptides were synthesized from Scilight-Peptide Inc. (Beijing, China) by the Fmoc solid-phase synthesis method. The unsophisticated peptides were purified using a Varian ProStar 218 HPLC instrument (Varian, Inc., Palo Alto, CA, USA) with an Aglient Venusil MP C18 column. Mobile phase A was 2% acetonitrile with 0.05% trifluoroacetic acid, and mobile phase B was 90% acetonitrile with 0.05% trifluoroacetic acid. Peptides were eluted with a linear gradient from 20% to 38% B in 18 min, at a flow rate of 1 mL/min. The separation was monitored at 220 nm using UV detection.

### 2.8. In Vitro Assays of ACE Inhibitory Activity

The ACE inhibitory activity of the samples was determined by the method of RP-HPLC [22]. The reaction system consisted of 20 μL ACE, 10 μL samples dissolved in borate buffer solution (BBS), and 20 μL substrate N-hippuryl-His-Leu hydrate (HHL). The enzyme activity was evaluated by product amount of hippuric acid (HA). BBS was used as the negative control, and captopril was used as the positive control. ACE inhibition (%) was calculated as follows:

ACE inhibition=(B−A)/B×100%

where A is the peak area of the sample, and B is the peak area of the negative control.

To determine the median inhibition concentration value (IC_50_) of samples, different concentrations of the sample were utilized to perform the ACE inhibitory activity, and IC_50_ was calculated by GraphPad Prism 9.5 [23].

### 2.9. Determination of ACE Inhibitory Pattern

The inhibition pattern of the candidate peptide on ACE was determined by the Lineweaver–Burk plot, according to Li et al. [24]. Briefly, ACE (1 mU) was incubated with different concentrations of peptide (0, 125 μM, and 250 μM) in reaction buffer, and then various concentrations of HHL (0.25, 1, 2, and 4 mM) were added separately. After reaction at 37 °C for 10 min, the inhibitory activity was determined as described above. The inhibition kinetics were analyzed using Graph Pad Prism software, in which the reciprocals of the substrate concentrations were the independent variable (x), and the reciprocals of the product formation rates were the dependent variable (y).

### 2.10. In Vivo Assay of Antihypertensive Effect in SHRs

Male spontaneously hypertensive rats (SHRs), 180 g, were provided by Charles River Laboratories (SCXK 2023-0011, Beijing, China). The animals were raised under the barrier system of Beijing University of Chinese Medicine and were allowed to freely eat and drink. All formalities were conducted according to the guidelines established by the Experimental Animal Center of Beijing University of Chinese Medicine, with approval number BUCM-2024012501-2138.

After one week of acclimation, the rats were randomly divided into 3 groups (*n* = 5): (1) blank group (ddH_2_O); (2) positive group (captopril, 10 mg/kg body weight/day); and (3) peptide group (ENWAAL, 20 mg/kg bodyweight/day). For the captopril dosage, the animal equivalent dose was calculated based on the clinical dose.

**Figure 1:**
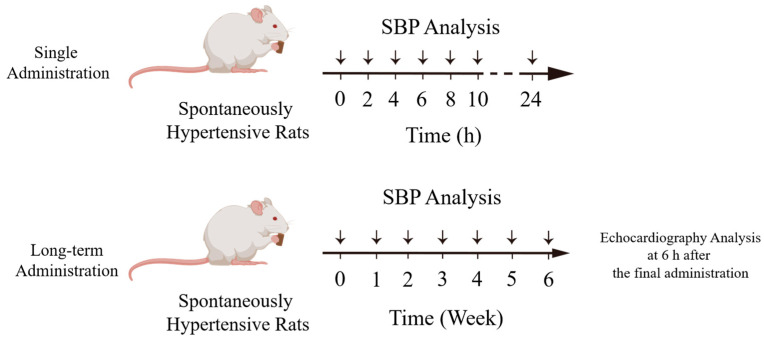
Schematic illustration of animal experiment.

The systolic blood pressure (SBP) of the rats was monitored by the noninvasive tail-cuff method using BP2000 (Visitech Systems, USA). For single administration (*n* = 5), SBP was measured at 0 h, 2 h, 4 h, 6 h, 8 h, 10 h, and 24 h post-administration. For successive administration (*n* = 5), ENWAAL was administered orally for 6 weeks, once a day, with a dose of 20 mg/kg body weight. The SBP was measured 6 h after administration once a week. A minimum of five consecutive readings were obtained. The maximum and minimum were removed to obtain the average blood pressure for single or long-term treatment (Figure 1).

### 2.11. Echocardiography Analysis

Six hours after the final administration, SHRs were anesthetized by 3% pentobarbital sodium (50 mg/kg) via intraperitoneal injection and fixed at a platform with supine position (Figure 1). The Vevo^®^ 2100 Ultra-High Resolution Small Animal Ultrasound Imaging System (Vevo SONIC, Toronto, ON, Canada) was employed for imaging with a short-axis view in M-mode. Parameters including the left ventricular anterior wall thicknesses in systole and diastole (LVAWs and LVAWd), left ventricular internal diameter in systole and diastole (LVIDs and LVIDd), left ventricular posterior wall thicknesses in systole and diastole (LVPWs and LVPWd), ejection fraction (EF), and fractional shortening (FS) were monitored.

### 2.12. Determination of Serum Ang II Content

The day after echocardiography analysis, SHRs were anesthetized via intraperitoneal injection of 3% pentobarbital sodium and subsequently euthanized by cervical dislocation. Blood samples were collected from aorta abdominalis and then centrifuged at 3000 rpm for 10 min to separate serum. The serum Ang II contents were measured using commercial ELISA KIT (mlbio, Shanghai, China).

### 2.13. Real-Time Quantitative PCR Analysis

Brainstems of each group were separated, rinsed in phosphate-buffered saline (PBS), and snap-frozen in liquid nitrogen for the analysis.

Total RNA was extracted from the tissue using TRIzol reagent (LABLEAD, Beijing, China). The quality and quantity of RNA were determined by spectrophotometry using ROCGENE Archimed X4. Reverse transcription was performed to synthesize cDNA with the First-Strand cDNA Synthesis Mix (F0202, LABLEAD, Bejing, China). Gene expression levels were subsequently analyzed by RT-qPCR using SYBR Green qPCR Master Mix Kit (E096, Novoprotein, Jiangsu, China). The amplification conditions were as follows: initial denaturation at 95 °C for 1 min, followed by 40 cycles of 95 °C for 15 s, and then 60 °C for 1 min. The relative expression of gene was calculated by the 2^−∆∆Ct^ method, with β-actin for normalization.

### 2.14. Western Blot Analysis

The protein expression levels of ACE and AT1R were determined by Western blot analysis. Total proteins were extracted using RIPA lysis buffer (P0013B, Beyotime, Shanghai, Chin), and the concentrations were determined by BCA assay. Protein samples were separated on 10% SDS-PAGE and then transferred to PVDF membrane. The membranes were incubated with anti-ACE (1:1000 dilution), anti-AT1R (1:1000 dilution), and anti-Tubulin (1:5000 dilution) antibody, sequentially, at 4 °C overnight. Then, further incubation in HRP-conjugated secondary antibody (1:5000 dilution) for 1 h at room temperature was conducted. Proteins were visualized using BeyoECL Star (Beyotime, Shanghai, China), and bands were analyzed with Image J software (Version 1.43).

### 2.15. Statistical Analysis

SPSS 26.0 was utilized to analyze the data. The data were presented as the mean ± SD. The *t*-test was employed to compare difference between two groups, and nonparametric statistics was used to compare multiple groups. A *p*-value less than 0.05 was considered statistically significant.

## 3. Results

### 3.1. Separation of ACE Peptides Fractions by Gel Filtration Chromatography

**Figure 2:**
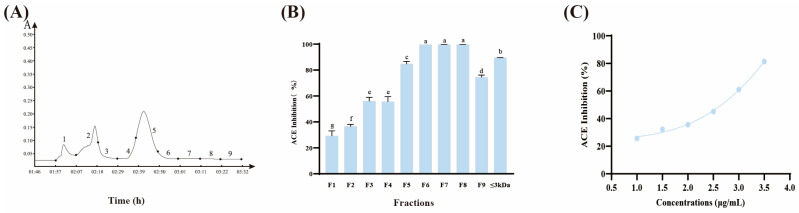
*Coix* hydrolysate purification and potency evaluation of ACE inhibitory activity. (**A**) Separation chromatogram of *Coix* hydrolysate using Sephadex G-10 column. The “A” shown on the *y*-axis represents absorbance at 220 nm. (**B**) ACE inhibitory activity of the separated fractions. (**C**) Dose–response curve for IC_50_ determination of Fraction F8. ^a–g^ Different letters indicate statistically significant difference (*p* < 0.05).

*Coix* glutelin hydrolysate with molecular ≤ 3 kDa was separated by gel filtration chromatography with Sephadex G-10 and collected at 10 min intervals. A total of nine fractions were obtained, which were designated F1–F9 (Figure 2A). The fractions were freeze-dried for the determination of ACE inhibitory activity in vitro, along with fraction ≤ 3 kDa, and the results are shown in Figure 2B. At the final concentration of 0.01 mg/mL for each sample, all the fractions displayed ACE inhibitory activity to some extent. Fraction F8 exhibited the highest inhibitory rate of 99.83%, versus the lowest of 29.29% for fraction F1. Then, different concentrations of the fraction F8 were prepared, and its IC_50_ was determined to be 2.65 μg·mL^−1^ (Figure 2C). Captopril was used as a positive control, showing an inhibitory activity of 98.43% under the same conditions.

### 3.2. Peptides Sequences of Determination

ESI-MS/MS was utilized to investigate the sequences of fraction F8. A total of 751 sequences were obtained. The molecular masses ranged from 0.8 kDa to 4.7 kDa with the animo acid number 7 to 47 (Table 1 and Figure S1).

### 3.3. Virtual Screening and Structurally Optimized of Peptides

The study of structure–activity relationship reveals that small molecule peptides generally exhibit high ACE inhibitory activity [21]. Therefore, a total of 62 biopeptides was selected for pharmacophore mapping analysis. As summarized in Table 2, Fit Values ranged from 0 to 0.97928. Among these, nine peptides exhibited a Fit Value equal to or greater than 0.9.

**Figure 3:**
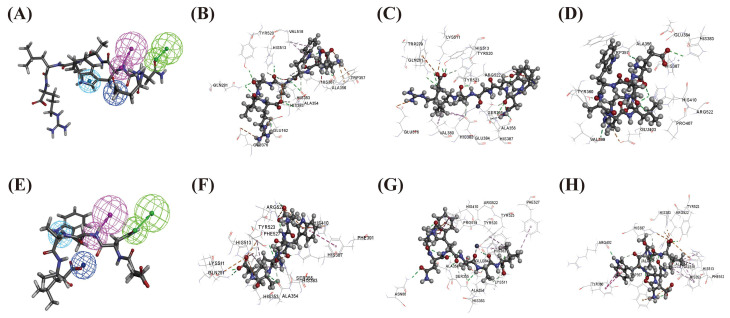
Pharmacophore model matching and the 3D interaction between compounds and ACE. Purple sphere indicates hydrogen bond donor, green sphere indicates hydrogen bond acceptor, light blue sphere indicates hydrophobic aromatic features, and dark blue sphere indicates negative ionizable. ENWAALR (**A**) and ENWAAL (**E**) matched with pharmacophore model. The binding modes of ENWAALR (**B**) and ENWAAL (**F**) with 1O86. The binding modes of ENWAALR (**C**) and ENWAAL (**G**) with 4CA5. The binding modes of ENWAALR (**D**) and ENWAAL (**H**) with 4BZR.

Molecular-docking results indicated that the EEAFVDDK, MTKPVEYR, KPILFSEK, GPFDVINK, ENWAALR, and QQEFFKSLYK (summarized in Table 2) achieved higher docking scores in 1O86, 4CA5, and 4BZR, which were higher than those of the original ligands. DYLADGPFEK and CFMSCSMPR showed binding capability only to 1O86 and 4CA5 (Figure 3A–D; Figure S3). ENWAALR, with a molecular weight of 858.95 Da, was the smallest one among the nine tested peptides Meanwhile, it contained an N-terminal hydrophobic glutamic acid, which is beneficial to promoting the inhibitory activity [25]. However, it showed weak binding to the ACE key animo acid, Ala354, and formed only limited hydrogen bonds and hydrophobic interactions. Therefore, ENWAALR was conducted for further structural optimization, and the mass spectrum for it is shown in Figure S2.

Following in silico proteolysis, the sequence ENWAAL exhibited a higher fit value than ENWAALR, indicating a more potential binding mode with ACE (Figure 3E–H and Figure S4; Table 2).

### 3.4. In Vitro ACE Inhibitory Activity of ENWAAL

**Figure 4:**
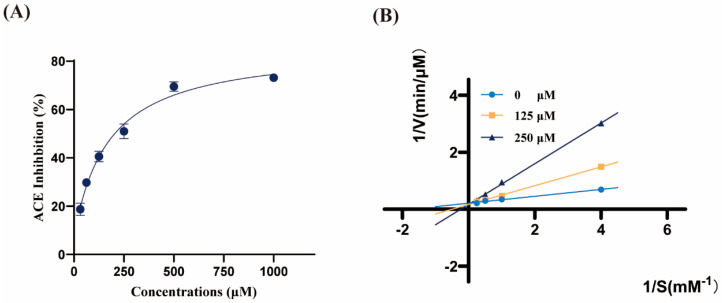
Inhibition of ACE by ENWAAL in vitro (n = 3, mean ± SD). (**A**) Nonlinear regression curve for the determination of IC_50_. (**B**) Lineweaver–Burk plot illustrating the competitive inhibition pattern. ACE inhibition was analyzed in the presence of different sample concentrations, as follows: 0 μM (●), 125 μM (■), and 250 μM (▴).

After being chemically synthesized, ENWAAL’s in vitro ACE inhibitory activity was evaluated by RP-HPLC. The inhibition rate for diverse concentrations of ENWAAL solutions was measured, and the IC_50_ was 210.57 ± 11.11 μM (Figure 4A).

### 3.5. The ACE Inhibition Pattern of ENWAAL

To investigate the inhibition pattern of ENWAAL on ACE, the Lineweaver–Burk plot method was employed. As shown in Figure 4B, the maximum reaction rate (Vmax) remained constant with the increasing peptide concentration, while the Michaelis constant (Km) was increased progressively. This finding suggested that ENWAAL functioned as a characteristic competitive ACE inhibitory peptide.

### 3.6. Antihypertensive Effect of ENWAAL in SHRs After Oral Administration

**Figure 5:**
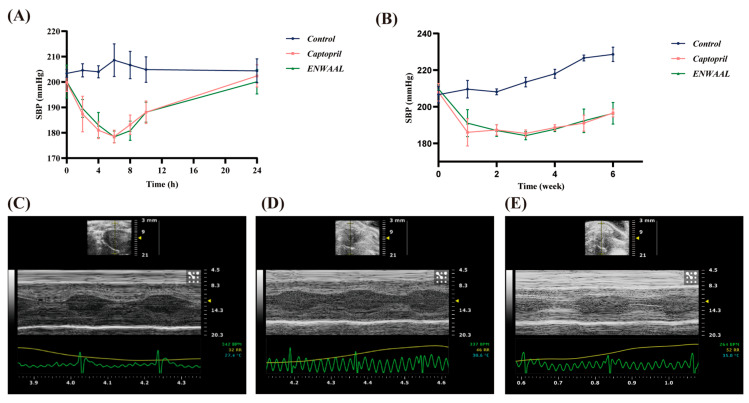
Antihypertensive effects of ENWAAL and its impact on cardiac function in SHRs (n = 3, mean ± SD). (**A**) The antihypertensive effect for single administration. (**B**) The antihypertensive effect during long-term oral administration. Echocardiographic assessment of cardiac function and structure across control (**C**), captopril (**D**), and ENWAAL (**E**) groups. LVAWs, LVAWd, LVIDs, LVIDd, LVPWs, LVPWd, ES, and FS were determined in echocardiographic assessment.

The antihypertensive effects of ENWAAL were evaluated in vivo in SHRs. For the single administration, the change in SBP within 24 h is indicated in Figure 5A. Compared to the control group, administration of ENWAAL resulted in a significant reduction in SBP. The SBP began to decline 2 h post-gavage, and it reached its lowest point at 6 h (178.4 ± 2.3 mmHg), which was 21.8 mmHg lower than pre-administration (*p* < 0.05). A similar antihypertensive effect was observed in the captopril group.

The effect of long-term oral administration of ENWAAL on SHRs was further investigated. The control group showed a steady increase in SBP, while ENWAAL treatment significantly lowered SBP during the 6 weeks (Figure 5B). All SBP values in the captopril group also paralleled those of the peptides group. At the end of the administration, the SBP of ENWAAL group and blank control group was 196.4 mmHg and 228.6 mmHg, respectively. These data indicated that ENWAAL exhibited a significant antihypertensive effect.

### 3.7. The Effect of ENWAAL on SHR Cardiac Function

The effects of ENWAAL on the cardiac structure and function were analyzed by echocardiography (Figure 5C–E; Table 3). LVAWd and LVAWs of blank group were 2.52 mm and 3.94 mm, respectively. Significant decreases for the ENWAAL group were detected (*p* < 0.05), i.e., 18.25% and 32.49% lower than the control group. EF was significantly increased (*p* < 0.05). The FS value was 12.46% higher than that of the control group. The results showed that ENWAAL could effectively alleviate cardiac remodeling and improve cardiac function.

### 3.8. Effect of ENWAAL on Serum Ang II Levels in SHRs

**Figure 6:**
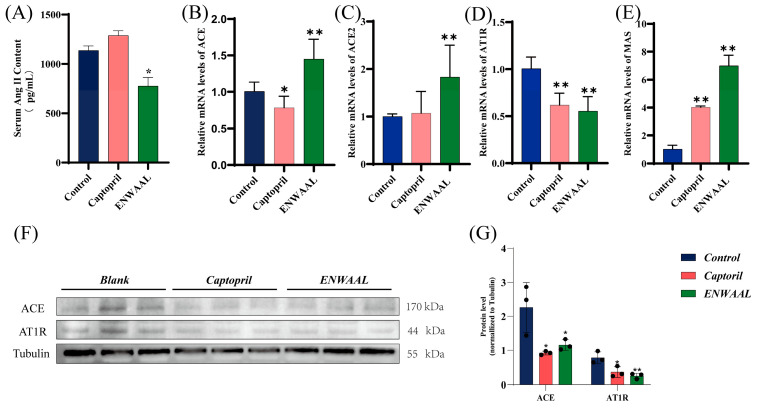
Serum Ang II content, RT-qPCR, and Western blotting analysis (n = 5, mean ± SD). (**A**) Serum Ang II content in SHRs. (**B**) *ACE*, (**C**) *ACE2*, (**D**) *AT1R*, and (**E**) *MAS* expression levels measured by RT-qPCR. (**F**) Representative images of Western blot assay of ACE and AT1R. (**G**) The analysis of WB light density performed for ACE and AT1R. * *p* < 0.05; ** *p* < 0.01 was compared with control group. Original images of (**F**) can be found in Supplementary Materials.

After 6 weeks of oral administration, the serum Ang II content of SHRs was determined. The results showed that the content of Ang II in the ENWAAL group was significantly decreased compared with the control group (Figure 6A), indicating that ENWAAL could downregulate the expression of serum Ang II in SHRs.

### 3.9. Effects of ENWAAL on RAS Genes and Proteins Expression in SHR Brainstem Tissue

Long-term oral administration of ENWAAL induced the expression alteration of brain RAS in both the ACE/Ang II/AT1R and ACE2/MAS axis. At the mRNA level, the expression of *AT1R* significantly decreased compared to that of the control group (*p* < 0.05). Regarding the ACE2/Mas axis, a significant increase was detected for these two genes, which were responsible for vessel vasodilatation (Figure 6B–E).

In order to ascertain the regulatory effect of ENWAAL on the brain RAS, the protein expression levels of ACE and AT1R were further evaluated. Compared with the blank control group, the ENWAAL treatment decreased the protein expression of ACE (49.51%), similar to the effect of captopril. Meanwhile, a similar tendency was detected for AT1R protein expression at the same time (Figure 6F,G).

## 4. Discussion

Enzymatic hydrolysis is the most common approach to generating biopeptides, with the advantages of mild conditions, high safety, and high biological activity [26]. A large number of bioactive peptides have been produced using gastrointestinal enzymes, usually pepsin, trypsin, and chymotrypsin. Trypsin has been shown to specifically cleave peptide bonds at the carboxyl side of Arg and Lys residues; therefore, more reproducible peptides may be generated by this enzyme [27]. Through enzymatic digestion by trypsin, plenty of ACE inhibitory peptides have been obtained so far. The protein of *Nannochloropsis oculata* was hydrolyzed by different enzymes, and trypsin hydrolysate exhibited the highest ACE inhibitory activity, and octapeptide GPGPFTVF was identified with an IC_50_ value of 39.77 μM [28]. LRAKA (LR-5) derived from the *Chlorella pyrenoidosa* trypsin hydrolysate had an IC_50_ of 36.19 μM [29]. YSK was obtained from the rice-bran protein trypsin digestion product with high ACE inhibitory activity (IC_50_ of 76 μM) [30]. ACE inhibitory peptide KAKP was identified from trypsin hydrolysate of pistachio of *Pistacia vera* L. through multiple purifications [31]. In this research, trypsin hydrolysates of *Coix* glutelin showed diverse ACE inhibitory activity, in which faction F8 ranked first, with an inhibition rate of 99.83%. Furtherly, EMWAAL was obtained from it with great antihypertensive effect.

Virtual screening, generally classified as ligand-based (LBVS) and structure-based (SBVS) approaches, could identify active compounds in the large chemical library to reduce time and cost [32]. Su et al. [33] used virtual screening to obtain ACE inhibitory peptides SFYYGK, RLVPVPY, and YIGNNPAKG from thin-leaf fine hairy mustard protein hydrolysate, which interacted with ACE through hydrogen bonding, electrostatic forces, van der Waals forces, and hydrophobic interactions. Li et al. [19] used ACE pharmacophore and receptor proteins to dock with *Coix lacryma-joib* peptides and obtained potent sequence GAAGGAF among the candidates. Zhang et al. [34] applied virtual screening and in silico hydrolysis to obtain a highly active antihypertensive peptide, FGSF, from vinegar black bean protein hydrolysate. In this experiment, candidate sequence ENWAALR was screened among 751 sequences by using a combination of LBSV and SBVS approaches, which had greatly improved the screening efficiency. Slightly imperfectly, ENWAALR demonstrated weak binding to the key amino acid residue, Ala 354, and failed to form sufficient hydrogen bonds and hydrophobic interactions. Therefore, it was further optimized by in silico hydrolysis. The subsequence ENWAAL took the advantages of smaller molecular weight. Furthermore, structure–function relationship has shown the N-terminal of the active peptides is mainly composed of aliphatic animo acids, which form interactions with ACE [24].

Hypertension can cause target organ alterations, especially in the heart, kidney, brain, and vasculature [35]. Hyperactivation of AT1R and ACE signaling in neurons exacerbates cognitive impairment, cell death, and inflammation [36]. AT1R is highly expressed in the endothelial and smooth muscle cells of cerebral arteries, microvessels, and capillaries, where it regulates cerebral blood flow, autoregulation, and blood–brain barrier integrity [37]. Currently, drugs can exert various pharmacological effects by regulating the neural RAS axis. Lu et al. discovered that pyridostigmine can inhibit the levels of ACE, Ang II, and AT1R in the paraventricular nucleus; enhance the expression of AT2R; and suppress RAS-induced oxidative stress and inflammatory responses in the paraventricular nucleus [38]. Microinjection of femtomole amounts of Ang- (1–7) in the NTS produced significant reductions in the blood pressure of urethane-anesthetized rats [39]. Similar to Ang- (1–7), central alamandine infusion improves baroreflex sensitivity for heart rate control. Alamandine also elicited site-specific cardiovascular effects by acting at MrgD within the CVLM and RVLM to produce vasodilation and decrease blood pressure [40]. However, limited evaluations have been conducted on the ACE inhibitory peptides regarding their regulation effects on the brain. In this experiment, the serum Ang II content of SHR was significantly reduced, and the protein expression levels of ACE and AT1R in brainstem tissues were significantly decreased, indicating that ENWAAL exerts antihypertensive effects by downregulating the ACE-Ang II-AT1R axis of both circulation and the neural RAS. Interestingly, there was an inconsistency between ACE transcription expression and its protein expression. At the mRNA level, the expected tendency of decreased expression was not obtained. The level of transcription may not be in line with the protein abundance, since post-transcriptional regulation, translational efficiency, and protein degradation may also influence the final protein expression levels. Given that proteins are the functional executors, they act as the ultimate determinant. Therefore, the reduced protein expression of ACE after ENWAAL treatment may ultimately contribute to the observed antihypertensive effect.

Although the precise exposure, bioavailability, and tissue distribution of ENWAAL remains unknown, the observed modulation of RAS pathway suggests that ENWAAL reached effective systemic levels after oral administration. Peptides can directly cross the blood–brain barrier after oral administration, despite the low bioavailability [41]. Meanwhile, peptides can also exert regulatory effects on the central nervous system through antioxidant effects, anti-inflammatory effects, and regulation of the gut–brain axis [42,43]. Nevertheless, there still remains a gap between present experiment results and the clinical translation. Safety evaluations and research on its efficacy using other administration methods, such as intravenous injection, and other animal models should be conducted in the future. Furthermore, to facilitate its clinical translation, formulation strategies such as nano-emulsions could be employed to enhance its in vivo delivery and pharmacological efficacy.

## 5. Conclusions

A novel peptide, ENWAAL, was generated from *Coix* hydrolyzation by chromatographic separation, virtual screening, and in silico proteolysis. This peptide interacted with ACE through competitive mode, with an IC_50_ value of 210.57 μM. Furthermore, ENWAAL demonstrated notable antihypertensive effects in SHRs greatly related to the ACE/Ang II/AT1R axis. Our discovery will provide a novel strategy for the management of hypertension, with multiplied efficiency in mitigating hypertension-induced organ injury, especially for mild-to-moderate hypertension.

## Figures and Tables

**Table 1:** The distribution of amino acid numbers of peptides in the F8 component.

Acid Numbers	≤10	11~15	16~20	>20
Numbers of peptides	62	255	202	232

**Table 2:** The predicted results of peptides by pharmacophore and docking.

Peptides Sequence	Fit Value	-CDOCKER ENENRGY (kcal mol^−1^)		
		1O86	4CA5	4BZR
Lisinopril	-	106.29		
Phosphopeptide FI	-		102.32	
K-26	-			156.91
DYLADGPFEK	0.979	227.090	185.553	NA
EEAFVDDK	0.976	249.713	197.899	248.316
GHDYIIVK	0.976	175.579	NA	187.305
MTKPVEYR	0.973	163.419	148.945	188.302
KPILFSEK	0.933	164.781	152.986	169.969
GPFDVINK	0.929	180.035	176.232	166.421
ENWAALR	0.922	164.555	157.778	171.240
QQEFFKSLYK	0.921	181.126	218.286	226.943
CFMSCSMPR	0.902	145.332	139.900	NA
Post in silico proteolysis of ENWAALR				
ENWAAL	0.953	159.286	142.800	161.353
ENWAA	0.923	160.872	126.175	164.753
AALR	0	114.266	99.125	98.935
ENW	0.961	146.433	105.767	134.067
LR	0	80.826	65.010	77.666

**Table 3:** Echocardiographic parameters from SHR after long-term oral administration (n = 3, mean ± SD).

	Control	Captopril	ENWAAL
LVAW; d (mm)	2.52 ± 0.03	2.28 ± 0.20	2.06 ± 0.11 *
LVAW; s (mm)	3.94 ± 0.03	3.46 ± 0.20 *	2.66 ± 0.02 *
LVID; d (mm)	4.99 ± 0.12	3.69 ± 0.32 *	4.39 ± 0.20 *
LVID; s (mm)	1.88 ± 0.16	0.78 ± 0.17 *	1.66 ± 0.15
LVPW; d (mm)	2.75 ± 0.20	2.67 ± 0.31	2.37 ± 0.54
LVPW; s (mm)	4.25 ± 0.06	3.95 ± 0.15	3.63 ± 0.40
EF (%)	82.07 ± 1.03	94.70 ± 5.22 *	91.04 ± 3.57
FS (%)	50.74 ± 0.74	82.09 ± 2.70 *	63.20 ± 6.51 *

* *p* < 0.05 was compared with control group.

## Data Availability

The original contributions presented in this study are included in the article/Supplementary Material. Further inquiries can be directed to the corresponding authors.

## Supplementary Materials

The following supporting information can be downloaded at https://www.mdpi.com/article/10.3390/biom16060888/s1, Figure S1. Mass spectrum of fraction F8. The inset is an enlarged view of the mass spectrum of ENWAALR. The *y*-axis represents relative abundance, and the *x*-axis represents *m*/*z*. Figure S2. MS/MS spectrum of ENWAALR. The *y*-axis represents intensity, and the *x*-axis represents *m*/*z*. Figure S3. Molecular docking of ENWAALR; Figure S4. Molecular docking of ENWAAL; Figure S5. Original images of Figure 6F.

## Abbreviations

The following abbreviations are used in this manuscript:

SHR	spontaneously hypertensive rats
ACE	angiotensin-converting enzyme
ACEIs	ACE inhibitors
Ang	angiotensin
AT1R	Ang II type I receptor
BBS	borate buffer solution
BP	blood pressure
CNS	central nervous system
EF	ejection fraction
Enos	endothelia nitric oxide synthase
FS	fractional shortening
HA	hippuric acid
HHL	N-hippuryl-His-Leu hydrate
LT	lamina terminalis
LVAW; s and LVAW; d	left ventricular anterior wall thicknesses in systole and diastole
LVID; s and LVID; d	left ventricular internal diameter in systole and diastole
LVPW; s and LVPW; d	left ventricular posterior wall thicknesses in systole and diastole
NTS	nucleus tractus solitarius
PBS	phosphate-buffered saline
PVN	hypothalamic paraventricular nucleus
RAS	renin–angiotensin system
RVLM	rostral ventrolateral medulla
SBP	systolic blood pressure
SFO	subfornical organ
