# Supplementary material for: Antihypertensive Peptide ENWAAL Derived from Coix Glutelin and Its Effect on the Expression of SHR Renin–Angiotensin System
Source: Biomolecules. 2026 Jun 16;16(6):888. doi: 10.3390/biom16060888 (PMC13297009; doi:10.3390/biom16060888)
Supplement: Supplementary file 1 [file biomolecules-16-00888-s001.zip › biomolecules-4321123-supplementary.pdf]

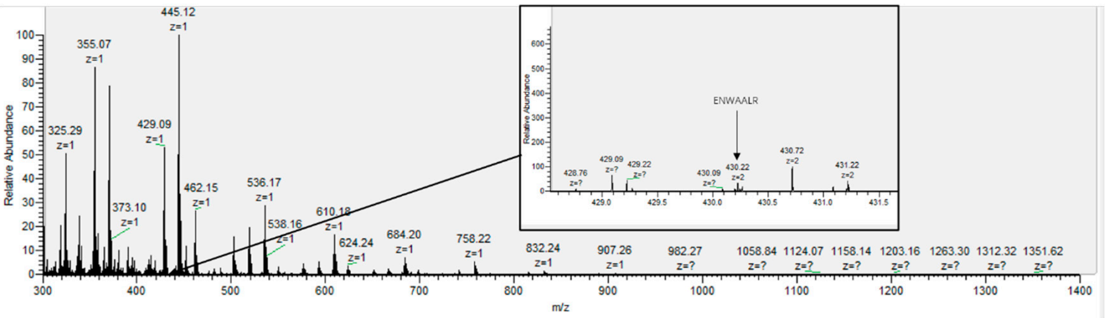


**Figure S1.** Mass spectrum of fraction F8. The inset is an enlarged view of the mass spectrum of ENWAALR. The y-axis represents relative abundance, and the x-axis represents m/z.

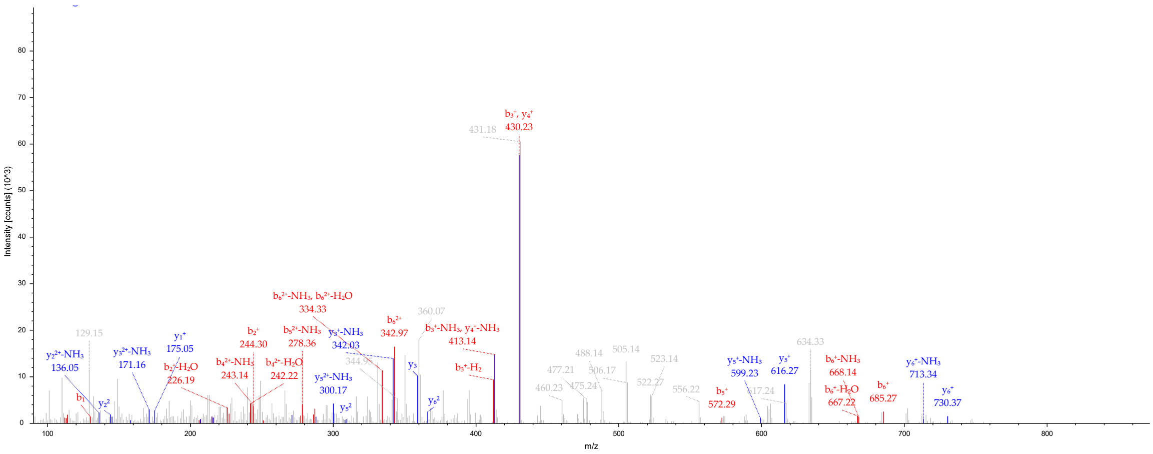


**Figure S2.** MS/MS spectrum of ENWAALR. The y-axis represents intensity, and the x-axis represents m/z.

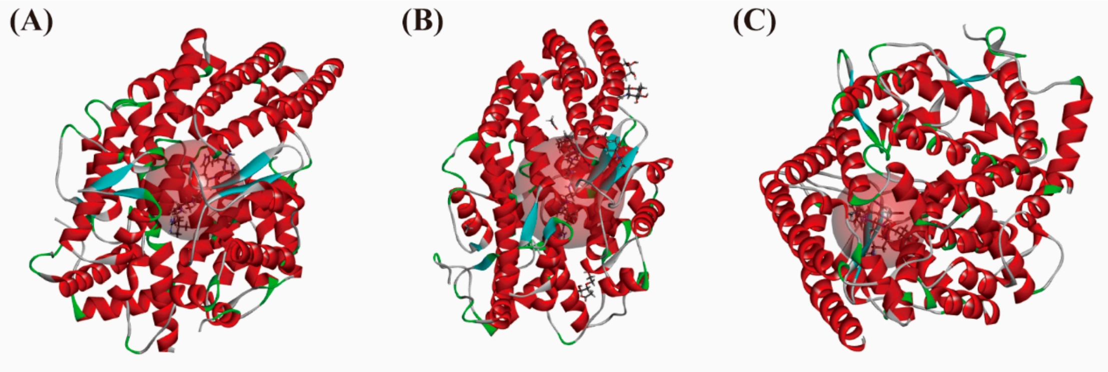


**Figure S3.** Molecular docking of ENWAALR. The binding modes of ENWAALR with 1O86(A). 4CA5 (B) and 4BZR (C).

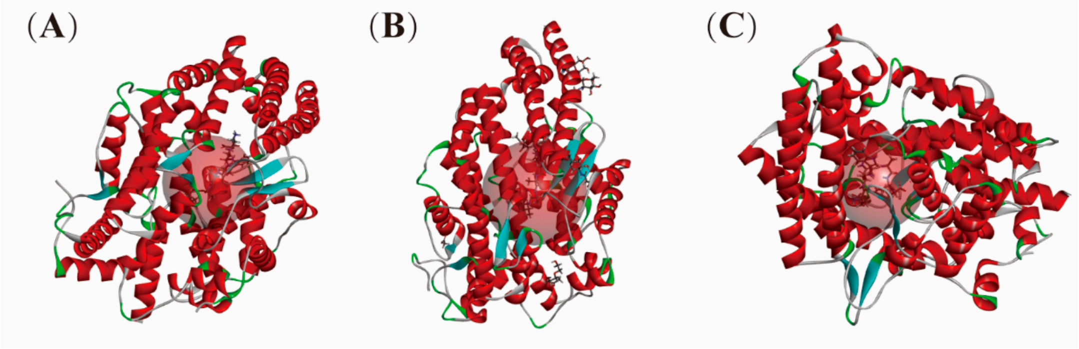


**Figure S4.** Molecular docking of ENWAAL. The binding modes of ENWAAL with 1O86(A). 4CA5 (B) and 4BZR (C).

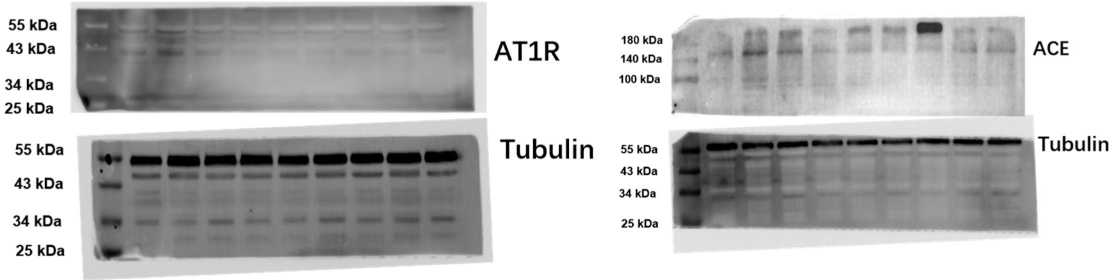


**Figure S5.** Original images of Figure 6F
