# Supplementary figures and images for: Antihypertensive Peptide ENWAAL Derived from Coix Glutelin and Its Effect on the Expression of SHR Renin–Angiotensin System
Source: Biomolecules. 2026 Jun 16;16(6):888. doi: 10.3390/biom16060888 (PMC13297009; doi:10.3390/biom16060888)

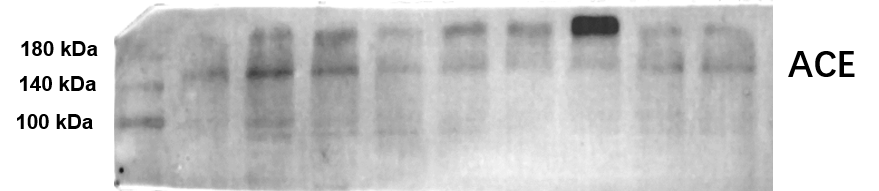

Supplement: Supplementary file 1 [file biomolecules-16-00888-s001.zip › biomolecules-4321123-original-images/ACE.png]

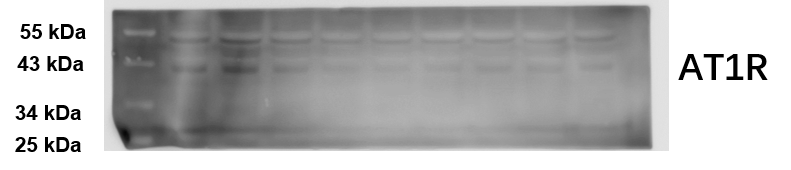

Supplement: Supplementary file 1 [file biomolecules-16-00888-s001.zip › biomolecules-4321123-original-images/AT1Rpng.png]

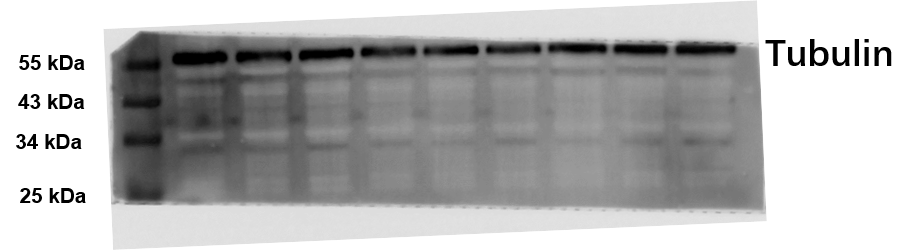

Supplement: Supplementary file 1 [file biomolecules-16-00888-s001.zip › biomolecules-4321123-original-images/Tubulin For ACE.png]

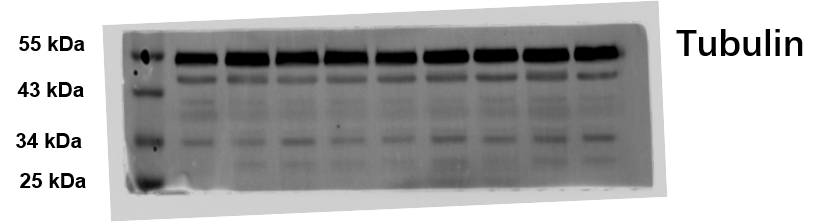

Supplement: Supplementary file 1 [file biomolecules-16-00888-s001.zip › biomolecules-4321123-original-images/Tubulin For AT1R.png]
